# A focused natural compound screen reveals senolytic and senostatic effects of *Isatis tinctoria*

**DOI:** 10.1080/19768354.2022.2143895

**Published:** 2022-11-11

**Authors:** Eun-Jung Kim, Jieun Woo, Seoungwoo Shin, HaeBeen Choi, Youngseok Kim, Junoh Kim, Chanhee Kang

**Affiliations:** a School of Biological Sciences, Seoul National University, Seoul, South Korea; b Biospectrum, Biospectrum Life Science Institute, Yongin, South Korea; c Center for Systems Gerosciences, Seoul National University, Seoul, South Korea; d Shinsegae International Inc., Seoul, South Korea

**Keywords:** Cellular senescence, senolytics, senostatics, *I. tinctoria*, skin aging

## Abstract

Natural products and their derivatives historically represent alternatives to conventional synthetic molecules for pharmacotherapy, ranging from cancer chemotherapeutics to cosmetic ingredients that exert anti-aging activities. Cellular senescence is considered a main driver of skin aging, yet natural products that target skin senescence in a specific manner are not thoroughly explored. Here, we performed a focused compound screen to identify natural products that exert anti-senescence effects. We found that *Isatis tinctoria*, woad extracts, displayed a senolytic effect on senescent human skin fibroblasts. Furthermore, treatment with woad extracts attenuated the expression of pro-inflammatory senescence-associated secretory phenotype (SASP), showing a senostatic activity. Intriguingly, woad extracts displayed only a marginal cytotoxic effect toward senescent human lung fibroblasts. Thus, our results reveal the potential activities of woad extracts for targeting skin senescence and suggest that woad extracts could be an attractive ingredient for cosmetics to prevent skin aging.

## Introduction

Cellular senescence is a stable arrest of cell cycle that can be triggered by various cellular damage, including telomere shortening, DNA damage, or oncogenic stresses (He and Sharpless 2017; Calcinotto et al. 2019; Gorgoulis et al. 2019). While senescent cells do not proliferate, they remain metabolically active, developing senescence-associated secretory phenotype (SASP). The SASP consists of numerous cytokines, chemokines, growth factors, and proteases that mediate pleiotropic effects of senescence, including paracrine senescence, tissue repair, immune surveillance, chronic inflammation, and tissue aging (Coppe et al. 2008; Coppe et al. 2010; Acosta et al. 2013; Kang et al. 2015; Birch and Gil 2020; Borghesan et al. 2020). Senescent cells accumulate with increasing aging in multiple tissues (Childs et al. 2015); transplanting senescent cells into mice is sufficient to cause persistent physical dysfunction and reduce survival (Xu et al. 2018). More importantly, genetic elimination of senescent cells improves several age-related pathologies and extends lifespan in mice (Baker et al. 2016). Since then, much effort has been made to pharmacologically alleviate the deleterious effects of senescence in two ways: (1) senolytics, agents that selectively kill senescent cells and (2) senostatics/senomorphics, agents that modulate the SASP and suppress its associated inflammation (Childs et al. 2017; Kirkland and Tchkonia 2017; Kang 2019; Kim EC and Kim 2019; van Deursen 2019; Kim J, Lee, Roh, et al. 2021). The first generation of senolytics is mostly repurposed from agents that treat cancer, which mainly target cell survival pathways, such as B-cell lymphoma 2 (BCL-2) family proteins and receptor tyrosine kinases (Zhu et al. 2015; Zhu et al. 2016). The second generation of senolytics leverages immunotherapies that are also employed for cancer treatment, including chimeric antigen receptor (CAR) T cells therapy (Amor et al. 2020). By contrast, most available senostatics target pathways involved in acute inflammation, lacking the specificity for senescence-associated inflammation (Kang 2019; Birch and Gil 2020; Wissler Gerdes et al. 2020).

Natural products (NPs) and their derivatives have significantly contributed to drug discovery, especially for cancer, metabolic syndrome, oxidative stress, and inflammatory diseases (Lee H et al. 2020; Park et al. 2020; Atanasov et al. 2021; Chen et al. 2021; Jang et al. 2021). Recently, it has been shown that a few natural ingredients, such as quercetin, fisetin, luteolin, and curcumin, exert senolytic activities in a context-dependent manner (Kang 2019; Birch and Gil 2020; Kirkland and Tchkonia 2020; Wissler Gerdes et al. 2020). For example, the plant flavonol quercetin, together with the tyrosine kinase inhibitor dasatinib, sensitized senescent preadipocytes and human umbilical vein cells (HUVECs) but had negligible senolytic effects on fibroblasts. Yet, fisetin mainly exerts senolytic effects on HUVECs but neither on fibroblasts nor on preadipocytes (Zhu et al. 2015). Accordingly, each NP senolytics has unique properties for ameliorating age-related pathologies (Kang 2019; Kim EC and Kim 2019; Birch and Gil 2020; Kim J, Lee, Roh, et al. 2021). Despite this recent advance, NPs specific for senolytic activities are not thoroughly explored yet. Thus, the crucial need for an additional search of senolytic and senostatic NPs is warranted.

*Isatis tinctoria*, also known as woad, is a short-lived perennial plant, mostly used as a traditional medicinal plant as well as a dye. Historically in many countries, woad has been utilized in treatment against measles, flu, cancer, and inflammatory diseases (Speranza et al. 2020). Biological activities of woad are mainly attributed to its anti-inflammatory, anti-viral, and anti-oxidant characteristics. No single compound has been isolated for such diverse bioactivities of woad yet; however, more defined extracts of woad were recently reported to have anti-inflammatory properties in a mouse model of dermatitis and human keratinocytes (Lotts et al. 2020), suggesting the possibility that woad extracts may be effective for skin inflammation associated with senescence as well.

In this study, we performed a focused compound screen with NPs that have anti-inflammatory activities to identify novel senolytics and senostatics. We found that woad extracts displayed a concentration-dependent senolytic effect in human dermal fibroblasts by activating caspase-dependent apoptotic pathway. Furthermore, woad extracts significantly suppressed the expression of major SASPs, including IL6, acting as senostatics. Collectively, our study proposes a potential application of woad extracts to ameliorate skin senescence.

## Materials and methods

### Cell lines and senescence induction

Human neonatal dermal fibroblasts (HDFs; PCS-201-010™), human lung fibroblasts (IMR90), and human foreskin fibroblast cells (BJ) were obtained from the American Tissue Type Collection (ATCC). Cells were maintained in 3% O_2_ and cultured in Dulbecco's modified Eagle's medium (DMEM) supplemented with 15% fetal bovine serum (FBS), penicillin/streptomycin, and 0.1 mM nonessential amino acids. Senescence was induced by treatment with bleomycin (10 μg/ml for 24 h; Cayman Chemicals) or replicative exhaustion, as described previously (Kang et al. 2015; Lee Y, Kim, Jeon, et al. 2021; Lee Y, Kim, Kim, et al. 2021).

### A focused senolytic screen

Young and senescent HDFs were seeded at a density of 2 × 10^4^ cells on a 24-well plate and incubated with the indicated natural products for 3 days, followed by treatment with 0.1 mg/mL MTT solution for 3 h. The optical density was measured at 570 nm with a plate reader after treatment.

### Preparation of natural product extracts

*Isatis tinctoria* (woad), *Rosmarinus officinalis, Rosa, Prunus persica, Nelumbo nucifera,* and *Citrus unshiu Marcov* were purchased from samhong geonjae yag-eobsa (Seoul, Korea), and extracted with aqueous ethanol. To prepare the ethanolic extract, the raw material was extracted with 70% (v/v) ethanol at 80 °C for 3 h, and the raw material was removed using a filter paper (Advantec, No. 131 qualitative filter paper (3 μm)). Ethanol was removed via rotary vacuum evaporation (EYELA, Tokyo, Japan) and the extract was lyophilized (yield 11%). The final NP powder was dissolved in water. For senolytic assay, cells were induced to senescence by bleomycin and then further treated with either NP extracts or navitoclax (control) for 3 days. For senostaic assay, cells were induced to senescence by bleomycin and then further treated with woad extracts for 9 days with every 2 days of refreshment.

### Cell death assay

Cell death was analyzed by FACS using propidium iodide (PI) according to the manufacturer's instructions. CellTiter-Blue assay (Promega) was additionally used for cell death assay, according to the manufacturer's instructions.

### Quantitative RT-PCR

Total RNAs were extracted using Favorprep^TM^ Tri-RNA reagent (Favorgen), and cDNA was synthesized using ReverTra Ace® qPCR RT Master Mix (Toyobo) according to the manufacturer's instructions. Quantitative RT-PCR was performed with SYBR TOPreal^TM^ qPCR 2× PreMIX (Enzynomics). Gene expression data were normalized with GAPDH, and primer sequences used in this study are as follows: GAPDH Fwd, CCTGCACCACCAACTGCTTA; GAPDH Rev, GGCCATCCACAGTCTTCTGAG; IL1A Fwd, AGTGCTGCTGAAGGAGATGCCTGA; IL1A Rev, CCCCTGCCAAGCACACCCAGTA; IL6 Fwd, CACTGGCAGAAAACAACCTGAA; IL6 Rev, ACCAGGCAAGTCTCCTCATTGA.

### Western blotting and antibodies

Protein lysates were prepared and loaded on SDS/PAGE gel and transferred onto nitrocellulose membrane. Transferred proteins were subjected to immunoblotting with the indicated antibodies. The primary antibodies used in this study were as follows: anti-Vinculin (Sigma, 1:20000), anti-GAPDH (Santa Cruz, 1:1000), anti-Caspase-3 (Cell signaling, 1:1000), anti-cleaved Caspase-3 (Cell signaling, 1:1000), anti-PARP (Cell signaling, 1:1000), anti-cleaved PARP (Cell signaling, 1:1000), anti-BAX (Cell signaling, 1:1000), anti-4EBP1 (Cell signaling, 1:1000), anti-phospho-4EBP1 (Cell signaling, 1:1000), anti-p70 S6 Kinase (Cell signaling, 1:1000), anti-Phospho-p70 S6 Kinase (Thr389) (Cell signaling, 1:1000), anti-RELA (Santa Cruz, 1:1000), anti-phospho-RELA (S536, 1:1000) (Cell signaling), anti-interleukin 6 (R&D systems, 1:400). HRP-conjugated anti-mouse IgG and anti-rabbit IgG secondary antibodies were obtained from the Jackson ImmunoResearch Laboratories Inc.

### mTOR activity test

Cells were briefly washed with PBS and subsequently starved for the indicated time by incubating the cells in amino acid-free DMEM (Welgene, LM001-90) supplemented with 15% of amino acid dialyzed FBS (Gibco, 26400044). Cells were then stimulated for 10 min through replacement by DMEM supplemented with 15% FBS. Protein lysates were subjected to Western blotting analysis.

## Results

### A focused natural product screen identifies *I. tinctoria* (woad) extracts as potential new senolytics

**Figure 1.**
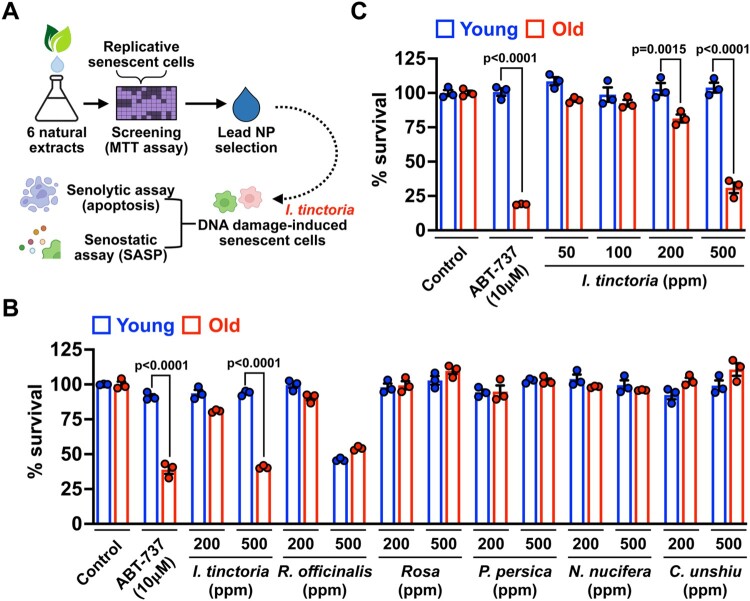
**A focused natural compound screen identifies *I. tinctoria* extracts as a novel senolytic agent** (A) Scheme of a focused natural compound screen for senolytics. (B) Young (PD 4∼10) and old (PD > 40) human dermal fibroblasts were treated with a total of 6 natural product extracts at 2 different concentrations (200 and 500 ppm) for 3 days. Cell viability was assessed by MTT assay and normalized to control. ABT-737 served as a positive control for senolytic activity. Data are mean ± SEM (n = 3), two-way ANOVA test. (C) Young (PD 4∼10) and old (PD > 40) human dermal fibroblasts were treated with *I. tinctoria* extracts at the indicated concentrations for 3 days. Cell viability was assessed by MTT assay and normalized to control. ABT-737 served as a positive control for senolytic activity. Data are mean ± SEM (n = 3), two-way ANOVA test.

In search of NPs that exert senolytic and senostatic effects, we selected a total of 6 NPs that are well-known to have anti-inflammatory activities (Atanasov et al. 2021). We employed a replicative senescence model of human dermal fibroblasts (HDFs) to examine whether treatment with NPs sensitizes them to cell death. Young and senescent HDFs were treated with two different concentrations of NPs [200 and 500 parts per million (ppm)] for 3 days, and cell viability was measured by assessing cell metabolic activity (MTT assay) (Figure 1A). Among tested, only *I. tinctoria* extracts (woad extracts) sensitized senescent HDFs over their young counterpart, which is comparable to a well-characterized senolytics, ABT-737 (Kang 2019; Borghesan et al. 2020; Wissler Gerdes et al. 2020) (Figure 1B). To further verify the senolytic activity of woad extracts, we tested senescent cell survival at their various concentrations. At 500 ppm, woad extracts displayed comparable senolytic effects to ABT-737 with no discernible toxicity on young cells (Figure 1C). Furthermore, woad extracts displayed a marginal senolytic effect at the lower level (200 ppm) (Figure 1C). Together, our focused NP screen identified woad extracts as potential new senolytics.

### Woad extracts exert senolytic effects toward skin fibroblasts but not lung fibroblasts

**Figure 2.**
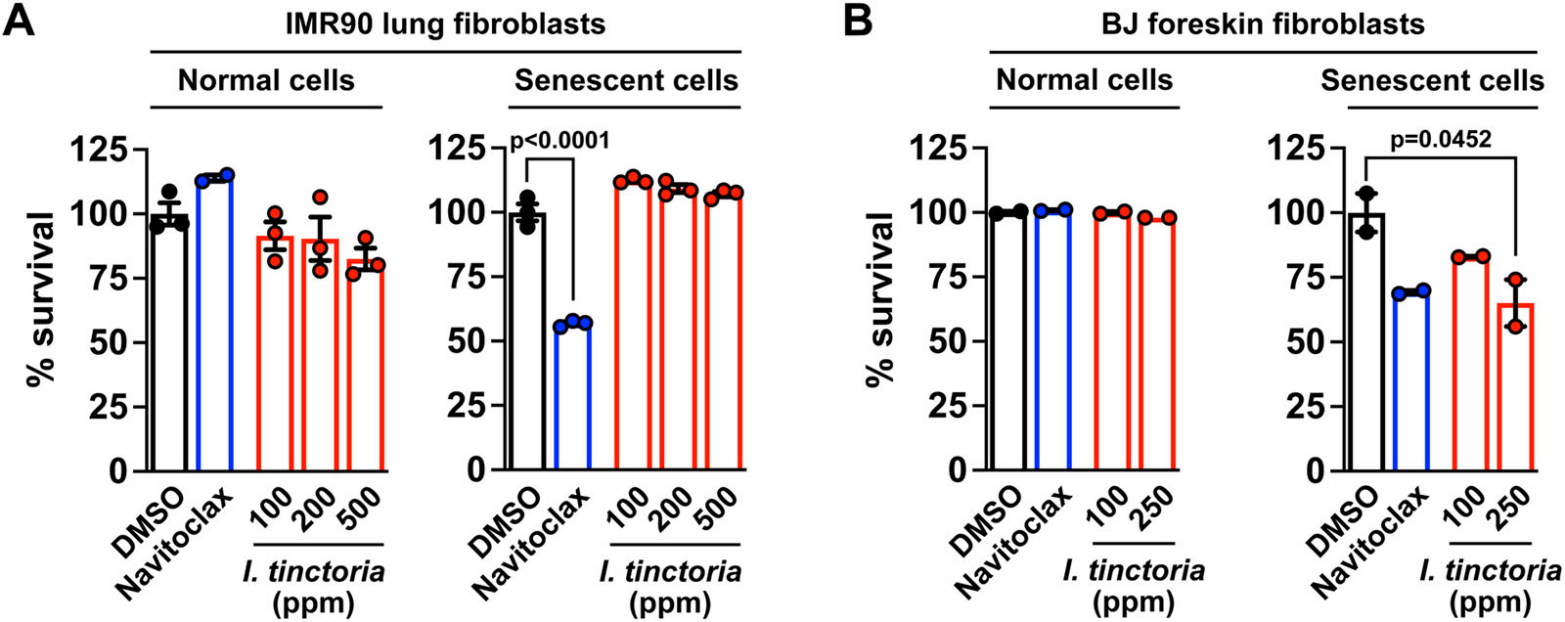
***I. tinctoria* extracts have a selective senolytic effect on human foreskin fibroblasts** (A) Human lung fibroblasts were treated with Bleomycin (10 μg / ml) for 24 h to induce senescence. Seven days later, normal and senescent IMR90 cells were treated with *I. tinctoria* extracts at the indicated concentrations for 3 days. Cell death was assessed by CellTiter blue analysis and normalized to DMSO. Navitoclax served as a positive control for senolytic activity. Data are mean ± SEM, one-way ANOVA test. (B) BJ cells were treated with Bleomycin (10 μg / ml) for 24 h to induce senescence. Seven days later, normal and senescent BJ cells were treated with *I. tinctoria* extracts at the indicated concentrations for 3 days. Cell death was assessed by PI staining. Navitoclax served as a positive control for senolytic activity. Data are mean ± SEM, one-way ANOVA test.

Senolytics and senostatics often act in a context-dependent manner: i.e. their efficacies vary depending on the types or origins of senescent cells (Kang 2019; van Deursen 2019; Wissler Gerdes et al. 2020; Kim J, Lee, Roh, et al. 2021). Thus, we decided to examine whether this is also the case for woad extracts. We employed two gold-standard models of senescent cell lines: senescent human lung and foreskin fibroblasts (IMR90 and BJ cells, respectively) induced by DNA damage (Kang et al. 2015; Lee Y, Kim, Jeon, et al. 2021; Lee Y, Kim, Kim, et al. 2021). We found that woad extracts selectively sensitized senescent BJ cells over their normal counterpart, which is comparable to the previously identified navitoclax (Zhu et al. 2016); however, woad extracts did not affect normal nor senescent IMR90 cells (Figure 2A and B), being consistent with results from our focused screen that utilized human dermal fibroblasts (Figure 1). Thus, woad extracts appear to be selectively senolytic for skin-origin fibroblasts. As senescent fibroblasts are a key source of *in vivo* senescence during skin aging (Campisi 1998), these data suggest that woad extracts may be available as selective senolytics for treating skin aging.

### Woad extracts induce caspase-dependent apoptosis in senescent skin fibroblasts

**Figure 3.**
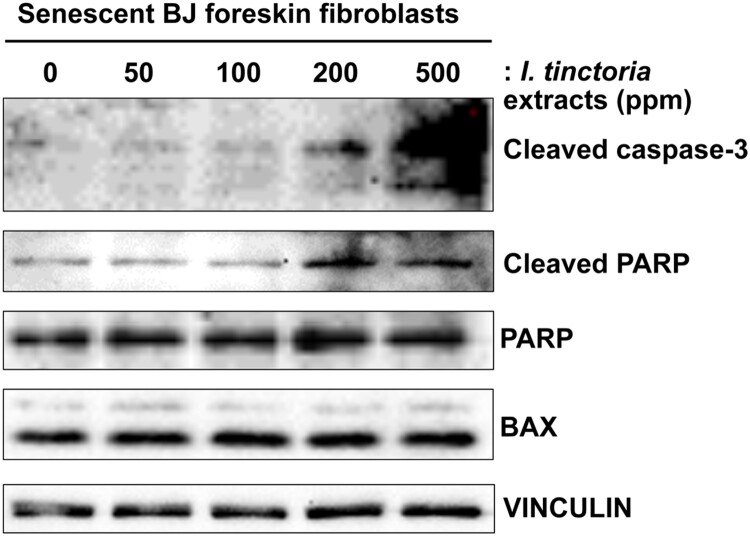
***I. tinctoria* extracts induce caspase-3-dependent apoptosis in skin senescent fibroblasts** BJ cells were treated with Bleomycin (10 μg / ml) for 24 h to induce senescence. Seven days later, normal and senescent BJ cells were treated with *I. tinctoria* extracts at the indicated concentrations for 3 days. The abundance of the indicated proteins was analyzed by Western blotting. Data are representative of two independent experiments.

To investigate the mechanism of action of woad extracts for their cytotoxic effects on skin fibroblasts, we examined multiple markers of apoptosis in senescent BJ cells upon treatment with woad extracts (Wissler Gerdes et al. 2020). First, we examined an active form of the executioner caspase-3, a cleaved caspase-3; we detected the increased levels of cleaved caspase-3 in senescent BJ cells upon treatment with woad extracts in a concentration-dependent manner (Figure 3). Next, we examined whether the activity of caspase-3 is indeed increased upon treatment with woad extracts by assessing the levels of cleaved poly (ADP-ribose) polymerase (PARP), a canonical substrate of caspase-3 (Galluzzi et al. 2018). Accordingly, we detected the increased levels of cleaved PARP upon treatment with woad extracts in a dose-dependent manner, verifying that caspase-dependent apopotosis pathway is activated in senescent skin fibroblasts by woad extracts. Lastly, we confirmed that treatment with woad extracts did not affect the expression of BCL2 associated X (BAX), a pro-apoptotic member of the BCL2 family proteins (Galluzzi et al. 2018). Collectively, our data suggest that woad extracts exert senolytic effects mainly through caspase-3-dependent apoptosis.

### Woad extracts suppress a subset of SASP expression at the post-transcriptional level in senescent skin fibroblasts

**Figure 4.**
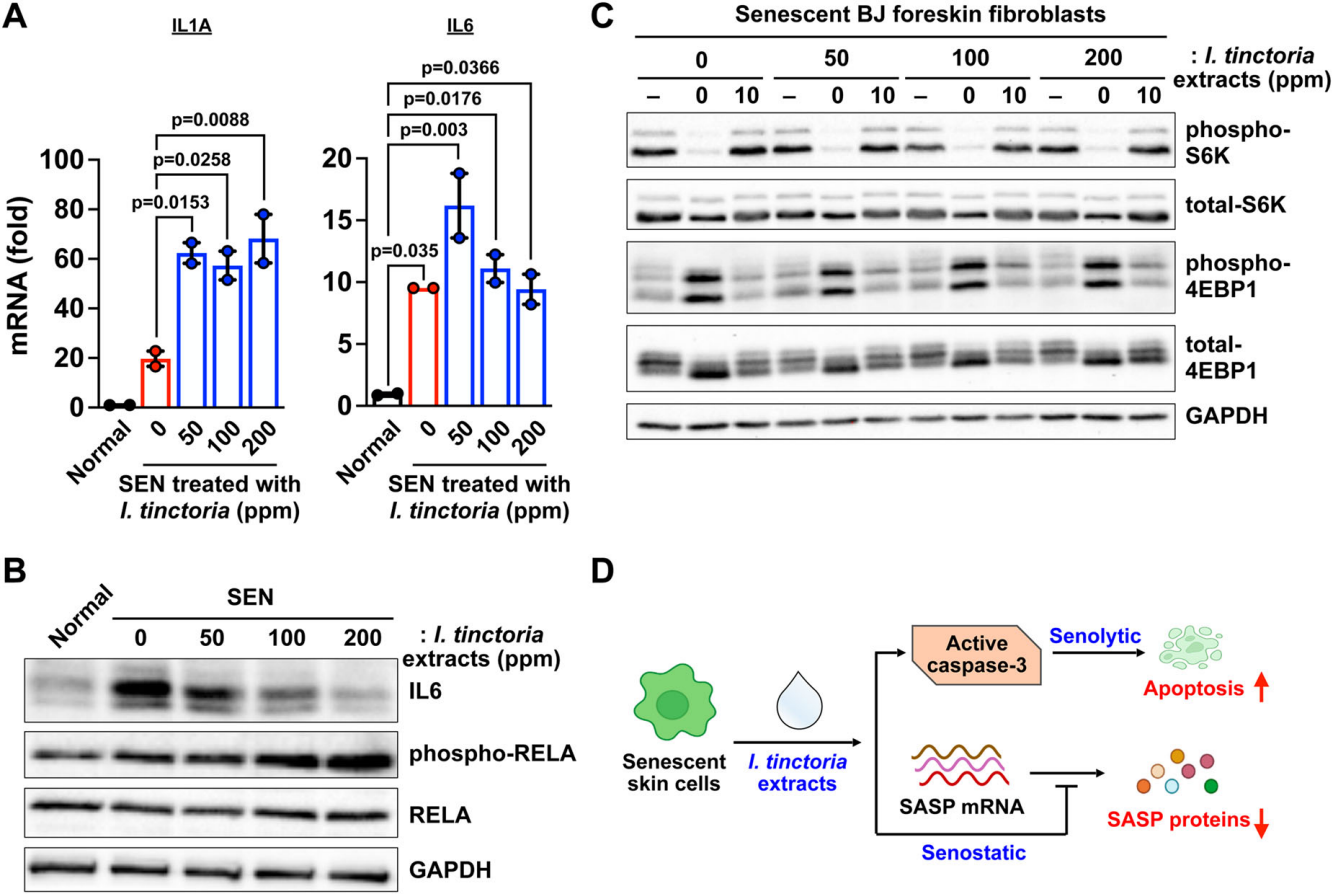
***I. tinctoria* extracts have a senostatic effect at the post-transcriptional level** (A) BJ cells were treated with Bleomycin (10 μg / ml) for 24 h to induce senescence. Normal and senescent BJ cells were treated with *I.tinctoria* extracts at the indicated concentrations for 9 days. The abundance of mRNAs for the indicated genes was quantified by RT-qPCR. Relative abundance of the indicated mRNAs is expressed as change with respect to expression in normal cells. Data are mean ± SEM (n = 2), one-way ANOVA test. SEN denotes senescent cells. (B) BJ cells were prepared as described in (A), and the abundance of the indicated proteins was analyzed by Western blotting. Data are representative of two independent experiments. (C) BJ cells were prepared as described in (A). For amino acid starvation and stimulation, cells were briefly washed with PBS and subsequently starved for the indicated time by incubating the cells in amino acid-free DEME supplemented with 15% of amino acid dialyzed FBS. Cells were then stimulated for 10 min through replacement by DMEM supplemented with 15% FBS. The abundance of the indicated proteins were analyzed by Western blotting. Data are representative of two independent experiments. (D) Model of how woad extracts display senolytic and senostatic effects. See text for details.

The best-characterized medicinal activities of woad extracts are anti-viral, anti-microbial, and anti-inflammatory activities (Lotts et al. 2020). As those processes are closely related to the SASP, we reasoned that woad extracts could impact SASP expression in senescent skin fibroblasts. Unexpectedly, we found that the expression of SASP genes rather increased at the transcriptional levels upon treatment with woad extracts in senescent skin fibroblasts (Figure 4A). These are consistent with the increased activity of the nuclear factor kappa B (NF-κB), as assessed by phosphorylation of RELA, a key component of the NF-κB that transcriptionally regulates the SASP (Chien et al. 2011; Kang et al. 2015) (Figure 4B). The SASP expression is known to be controlled at different layers of regulation, including transcription, epigenetic regulation, mRNA stability, translation, and protein stability (Herranz et al. 2015; Kang et al. 2015; Laberge et al. 2015; Tasdemir et al. 2016; Hernandez-Segura et al. 2018; Birch and Gil 2020; Sofiadis et al. 2021; Wiley et al. 2021). Thus, we examined the protein expression of IL6, a key inflammatory component of the SASP. We found that treatment with woad extracts significantly decreased IL6 protein abundance in senescent cells (Figure 4B), suggesting that woad extracts have senostatic activities by suppressing the SASP at the post-transcriptional level. Previous studies show that the mammalian target of rapamycin complex 1 (mTORC1) enhances SASP expression by modulating SASP translation as well as mRNA stability (Herranz et al. 2015; Laberge et al. 2015). Therefore, we examined whether treatment with woad extracts affect the activity of mTORC1 during senescence; treatment with woad extracts did not reduce the phosphorylation of ribosomal protein S6 kinase (S6K1) nor that of eukaryotic translation initiation factor 4E-binding protein 1 (4E-BP1) (Figure 4C), two well-known substrates of mTORC1 (Liu and Sabatini 2020). In conclusion, woad extracts act as senostatics by suppressing SASP expression at the post-transcriptional level in a mTORC1-independent manner.

## Discussion

Increasing evidence suggests that senescence is a common denominator of the aging process and contributes to a myriad of age-related pathologies, including atherosclerosis, osteoarthritis, metabolic syndrome, and cancer (Lopez-Otin et al. 2013; Childs et al. 2017; McHugh and Gil 2018; Borghesan et al. 2020). Therapies that attenuate the deleterious effects of senescence have been proposed to promote healthy aging by alleviating such age-related diseases and disabilities as a group (McHugh and Gil 2018; Gorgoulis et al. 2019; Kang 2019; Kim EC and Kim 2019; van Deursen 2019; Birch and Gil 2020; Wissler Gerdes et al. 2020; Kim J, Lee, Roh, et al. 2021). Senescence is also closely associated with skin aging (Ho and Dreesen 2021); therefore, senotherapy (i.e. senolytics and senostatics) could be effective to delay, prevent, and treat skin aging. In this study, we investigated a potential senotherapuetic efficacy of NPs, *Isatis tinctoria* (woad) extracts, in human skin fibroblasts.

The first generation of senolytics, including navitoclax and dasatinib, is mostly repositioned from agents that treat cancer and thus has similar limitations, such as side effects and toxicity. For example, navitoclax causes thrombocytopenia and neutropenia, greatly limiting its *in vivo* usage as senolytics (Zhu et al. 2015; Zhu et al. 2016; Kang 2019; Wissler Gerdes et al. 2020). NPs and their derivatives could provide a relatively safer option to conventional synthetic molecules (Karimi et al. 2015), being attractive alternatives for pharmacotherapy (Atanasov et al. 2021). Indeed, much attention has been made to searching NPs that target senescent cells, either by selectively sensitizing them to cell death (senolytics) or by suppressing senescence-associated inflammation (senostatics). Quercetin, fisetin, luteolin, and curcumin are currently considered promising NPs with senolytic or senostatic efficacies (Kirkland and Tchkonia 2020; Wissler Gerdes et al. 2020). As these NPs have context-dependent senolytic effects, future studies are warranted to examine whether they could be effective for skin senescence as well.

We propose woad extracts as a novel senolytic and senostatic agent potentially specific for skin senescence, as evidenced by their efficacy on two different types of human fibroblasts derived from skin. Woad extracts also exert such activity toward two different types of senescence models, replicative senescence and DNA damage-induced senescence; i.e. woad extracts could target either core senescence regulatory pathways, common in replicative and DNA damage-induced senescence. Alternatively, woad extracts may target the stress support networks of senescence, including redox homeostasis and proteostasis, which were recently shown to be critical to maintaining the homeostatic states of senescence (Kang and Elledge 2016; Kwon et al. 2017; Kim J, Lee, Jeon, et al. 2021; Kim J, Lee, Roh, et al. 2021; Lee Y, Kim, Kim, et al. 2021). Consistently, previous studies showed that woad extracts have anti-oxidative properties (Speranza et al. 2020); we also showed that woad extracts affect the expression of the SASP at the protein level. Furthermore, woad extracts have been shown to include multiple bioactive compounds, such as alkaloids, phenolic compounds, polysaccharides, glucosinolates, carotenoids, and fatty acids (Speranza et al. 2020). Some of these (e.g. luteolin and quercetin) were previously identified as senolytics that might be required for the senolytic activity of woad extracts (Kirkland and Tchkonia 2020). It remains elusive what ingredient(s) and which molecular mechanism contribute to the unique senotherapeutic properties of woad extracts.

Woad extracts have been widely used in cosmetic industries for their skin protective and anti-inflammatory activities (Speranza et al. 2020; Atanasov et al. 2021). Since skin senescence leads to dysregulated skin homeostasis and chronic inflammation (Campisi 1998), it is plausible to assume that woad extracts may modulate skin senescence. Our results for the first time demonstrate that woad extracts have senolytic and senostatic effects on skin fibroblasts in a selective manner, suggesting that pleiotropic biological activities of woad extracts may result from their senotherapuetic effects. It should be noted that our senescence model employed bleomycin as an inducer of DNA damage that might differ from the one induced by ultraviolet radiation, a major cause of skin aging (Amaro-Ortiz et al. 2014). Further *in vivo* studies in a more physiological condition will be necessary to consolidate this exciting hypothesis for cosmetic industries.

In summary, we have shown that woad extracts have distinct senotherapeutic effects over several NPs in human skin fibroblasts. Woad extracts cause caspase-3-dependent apoptosis in senescent cells as well as suppressing the SASP at the post-transcriptional level. Constant damaging stimuli, including UV exposure, cause skin senescence, which eventually exceeds the capacity of the immune system and leads to the accumulation of senescent cells, causing skin aging (Ho and Dreesen 2021). Thus, therapies that manage senescent cells can be a promising strategy for healthy skin aging. As many NPs are already used as anti-aging agents in cosmetic industries, it will be interesting to examine whether such NPs have senotherapeutic activities, similarly to woad extracts tested in this study.
